# Dysregulation of threat neurocircuitry during fear extinction: the role of anhedonia

**DOI:** 10.1038/s41386-021-01003-8

**Published:** 2021-04-08

**Authors:** Katherine S. Young, Susan Y. Bookheimer, Robin Nusslock, Richard E. Zinbarg, Katherine S. F. Damme, Iris Ka-Yi Chat, Nicholas J. Kelley, Meghan Vinograd, Marcelina Perez, Kelly Chen, Aileen Echiverri Cohen, Michelle G. Craske

**Affiliations:** 1 Department of Psychology, University of California, Los Angeles (UCLA), Los Angeles, CA USA; 2 Social, Genetic and Development Psychiatry (SGDP) Centre, Institute of Psychology, Psychiatry and Neuroscience, King’s College London, London, UK; 3 NIHR Maudsley Biomedical Research Centre, King’s College London, London, UK; 4 Department of Psychiatry and Biobehavioral Sciences, University of California, Los Angeles (UCLA), Los Angeles, CA USA; 5 Department of Psychology, Northwestern University, Evanston, IL USA; 6 The Family Institute at Northwestern University, Evanston, IL USA; 7 Temple University, Philadelphia, PA USA; 8 Department of Psychology, University of Southampton, Southampton, UK; 9 Center of Excellence for Stress and Mental Health, Veterans Affairs San Diego Healthcare System, San Diego, CA USA; 10 Department of Psychiatry, University of California San Diego, San Diego, CA USA; 11 Department of Psychology, University of Arizona, Tucson, AZ USA

**Keywords:** Emotion, Limbic system

## Abstract

Dimensional models of anxiety and depression highlight common and distinct symptom clusters that are thought to reflect disruptions in underlying functional processes. The current study investigated how functioning of threat neurocircuitry relates to symptom dimensions of anxiety and depression. Participants were aged 18–19 years (*n* = 229, 158 female) and were selected to ensure a range of scores on symptom measures. Symptom dimensions of “General Distress” (common to anxiety disorders and depression), “Fears” (more specific to anxiety disorders), and “Anhedonia-apprehension” (more specific to depression) were evaluated. Participants underwent functional magnetic resonance imaging during a Pavlovian fear conditioning paradigm. Multilevel modeling analyses estimated relationships between symptom dimensions and activation in threat neural circuitry. Exploratory whole brain analyses were also conducted. Threat-related neural activity was not associated with General Distress or Fears. Anhedonia-apprehension was associated with activation of bilateral amygdala, anterior insula and dACC during late extinction. We found no evidence to support an association between symptom dimensions of General Distress or Fears with threat circuitry activation in a large sample of young adults. We did, however, find that the symptom dimension of Anhedonia-apprehension was significantly associated with threat-related neural activation during fear extinction. This effect requires replication in future work but may reflect anhedonic impairments in learning when contingencies are altered, possibly linked to the rewarding relief of an unexpectedly absent threat.

## Introduction

Anxiety disorders are associated with a heightened tendency to acquire conditional fears and a reduced capacity to extinguish them [1]. Maladaptive fear learning is therefore considered a central mechanism in the development and treatment of anxiety disorders, and fear conditioning paradigms have been widely used to study these processes in the laboratory. Translational research on fear learning across human and animal studies converge on a threat neural circuit, including the amygdala, insula, hippocampus and ventromedial prefrontal and anterior cingulate cortices [vmPFC/ACC; 2, 3–6].

Neurobiological models suggest that anxiety disorders are driven by elevated threat responding, indicated by: (i) heightened reactivity in the amygdala, dorsal ACC (dACC) and insula to threat cues during conditional fear acquisition; (ii) impaired “safety signaling” in the vmPFC, reducing inhibition of threat responses during extinction and extinction recall; and (iii) impaired contextual encoding of memories in the hippocampus, leading to overgeneralization of fear acquisition [7–12]. This set of features is posited to underlie the onset, persistence and spread of fears [13]. However, empirical evidence from fMRI studies of fear conditioning is equivocal. With respect to fear acquisition, some studies have demonstrated associations between amygdala activation, insula or ACC and trait anxiety or symptoms of anxiety [e.g. 14–16], but two well-powered studies demonstrated no significant associations [10, 17]. A recent study reported differences in amygdala activation during extinction and dACC activation as a function of physiological arousal (skin conductance response) during extinction recall in persons with anxiety disorders and healthy controls [18]. Impaired vmPFC “safety signaling” has been observed in anxiety disorders, but the phases in which effects are found vary across studies (e.g., acquisition and recall [10]; extinction and recall [19]; extinction only [17]). Reduced hippocampal activation during acquisition has been demonstrated in some studies of anxiety disorders [e.g.^10^, ^19^] but not in others [e.g. 17].

Comorbidity between anxiety and depression [20] may contribute to inconsistent findings across studies. Neurobiological models of depression most commonly emphasize disruptions in reward circuitry [21]. However, there is also evidence for disruptions in threat circuitry (amygdala, ACC and vmPFC) to innately aversive stimuli (e.g. fearful or angry faces) in relation to depression [22–26] but almost no investigation of Pavlovian fear learning. In one study, the presence of comorbid depression was associated with decreased insula and dorsolateral prefrontal activation during fear conditioning relative to panic disorder alone [14].

Comorbidity between diagnostic entities is partly explained by shared dimensional features (e.g., negative affect [27]). Dimensional models of anxiety and depression offer greater precision in elucidating underlying neural processes [28]. Studies to date have considered only single dimensions (e.g., trait anxiety or physiological arousal) that are relatively specific to anxiety disorders vs. depression. Here, we evaluate a comprehensive dimensional model of anxiety and depression, the trilevel model [29, 30]. The trilevel model identifies one broad and two intermediate symptom dimensions that represent variance shared across anxiety and depression: “General Distress” (broad) is common to anxiety and depression; “Fears” (intermediate) is more specific to anxiety; and “Anhedonia-apprehension” (intermediate) is more specific to depression. Variance associated with individual disorders (e.g., panic disorder, social anxiety disorder etc.) is represented by “narrow” symptom clusters in the trilevel model. As neurobiological models point towards a common disruption to threat circuitry across anxiety disorders [7–12], we focus upon the broad and intermediate trilevel dimensions.

The current study examined associations between dimensional symptom measures and activation of threat neural circuitry during Pavlovian fear conditioning. Participants were young adults (recruited as part of a longitudinal study) who were selected to ensure a range of anxiety and depression symptoms. Based on neurobiological theories of anxiety reviewed above, we hypothesized that activation of threat neural circuitry during fear learning would be more strongly associated with General Distress and Fears symptom dimensions, both strongly associated with anxiety, than with Anhedonia-apprehension, more strongly associated with depression. We predicted that General Distress and Fears would be: (i) positively associated with the magnitude of amygdala, insula, and ACC activation during fear acquisition (CS+ > CS−); (ii) negatively associated with the magnitude of vmPFC activation across fear acquisition (CS+ > CS−), extinction (CS+ E > CS−), and recall (CS+ U > CS+ E); (iii) negatively associated with hippocampus activation during fear acquisition (CS+ > CS−).

## Methods and materials

### Participants

Participants were recruited for the Brain, Motivation, and Personality Development (BrainMAPD) study at the University of California, Los Angeles and Northwestern University, which investigated depression and anxiety in late adolescence and early adulthood. Participants were 272 individuals aged 18–19 years (182 female, mean age = 19.16 years, *SD* = 0.52). They were selected from a larger screening sample of 2461 individuals to represent a broad range of scores on self-reported trait neuroticism and reward sensitivity to maximize variance in threat- and reward-related sensitivity (see Supplementary Materials for details). Exclusion criteria were: lack of right-handed dominance, not fluent in English, traumatic brain injury, MRI contraindications, pregnancy, color blindness, lifetime psychotic symptoms, bipolar I disorder, clinically significant substance use disorder in the past 6 months, and antipsychotic medication usage. Participants provided written, informed consent and all procedures were approved by the IRB at each institution.

Of this group *n* = 229 (158 female) are included in analyses for fear acquisition, *n* = 220 (151 female) are included for fear extinction, and *n* = 212 (142 female) for extinction recall (Table 1, different sample sizes were mostly due to exclusions based on excessive motion in the scanner, see Supplementary Materials for full details). In total, 260 individuals contributed data to one or more task phases, of whom 223 completed SCID-5 interviews. Fifty-six participants (21.53%) met criteria for a current anxiety disorder, but no depressive disorder; 18 (6.92%) met criteria for current anxiety and depressive disorders; and three (1.15%) met criteria for a depressive disorder but no anxiety disorder.

**Table 1:** Demographic factors and symptom dimension scores of participants compared across scanning site.

	UCLA	Northwestern	Statistic	*p* value
N	115	145		
Sex (N, %)			*X*^2^ = 1.05	0.31
Female	77 (67.0%)	87 (60.0%)		
Male	38 (33.0%)	58 (40.0%)		
Age (M, SD)	19.03 (0.51)	19.18 (0.50)	*t* = 1.98*	0.049
Ethnicity				
Not Hispanic/Latino	81 (70.4%)	111 (76.6%)	*X*^2^ = 0.95	0.33
Hispanic/Latino	34 (29.6%)	34 (23.4%)		
Race (N, %)			*X*^2^ = 27.25*	<0.001
White	61 (53.0%)	80 (55.2%)		
Asian	46 (40.0%)	27 (18.6%)		
Black	4 (3.5%)	17 (11.7%)		
Native American	1 (0.9%)	3 (2.1%)		
Multiracial	2 (1.7%)	18		
Declined to report	1 (0.9%)	0 (0.0%)		
Psychotropic medication use (N, %)	4 (3.5%)	21 (14.5%)	*X*^2^ = 7.72*	0.005
Symptom dimension scores (M, SD)				
General distress	−0.04 (0.94)	0.11 (0.91)	*t* =1.24	0.215
Fears	0.07 (0.93)	−0.09 (0.78)	*t* = −1.55	0.123
Anhedonia-apprehension	0.12 (0.85)	−0.13 (0.95)	*t* = 1.77	0.079

Participants were marginally significantly younger at UCLA than at Northwestern University; the racial identity of individuals across sites was significantly different, with a higher proportion of Asian participants and UCLA and a higher proportion of Black participants at Northwestern University.

*p < 0.05.

### Symptom assessment and factor analysis

Immediately prior to MRI scans, participants completed questionnaire measures of anxiety and depression, to generate hierarchical^1^ tri-level model factor scores [29, 30]. These included items from: Fear Survey Schedule-II [31], Albany Panic and Phobia Questionnaire [32], Self-Consciousness subscale of the Social Phobia Scale [33, 34], Inventory to Diagnose Depression [35], Mood and Anxiety Symptom Questionnaire [36], Penn State Worry Questionnaire [37], and Obsessive Compulsive-Inventory Revised [38]. Confirmatory factor analyses (CFA) demonstrated goodness of fit of the tri-level hierarchical model [identified in prior work; 29, 30] to the data collected in the present study (see details reported in Kramer et al. 2020 [39]). Factor estimates from this model were saved and used to represent symptom dimensions of General Distress, Fears, and Anhedonia-apprehension. (Note: the Anhedonia-apprehension factor is largely driven by positive affect items (e.g., reverse-scored items such as: “felt like I was having a lot of fun”, “felt really happy”), which have a standardized loading average magnitude of 0.71 whereas the strongest standardized loading of an apprehension item (e.g., “feeling discouraged about the future”, “feeling pessimistic about the future”) has a magnitude of only 0.28).

### Fear conditioning paradigm, skin conductance, and contingency awareness

Participants completed a differential Pavlovian Fear Learning Task [40, 41], conducted over two scanning sessions (*M* = 2.76 days apart, *SD* = 2.48) in three phases: acquisition, extinction (session 1), and recall (session 2). During acquisition, participants viewed two CS+ images and one CS− image (rooms with different colored lights). There were eight trials of each CS+ (16 trials total), 62.5% of which were reinforced with a US (electric shock), and 16 CS− trials. During extinction, participants viewed 16 CS+ trials with no US (extinguished CS+ , termed CS+ E) and 16 CS− trials. During recall, participants viewed 8 CS+ E trials, 8 trials of the CS+ not viewed during extinction (“unextinguished CS ”,+  CS+ U) and 16 CS− trials. Each trial consisted of a 3-second “context” image (image of room with no light), followed by a 6-second CS image (room with light). The inter-trial-interval varied from 12–15 sec. Galvanic skin conductance was recorded from electrodes on the left index and middle finger throughout the task. After data exclusion based on signal quality and motion artifacts, there were *n* = 218 skin conductance datasets available for analysis (see Supplementary Materials for details). At the end of acquisition and extinction, “contingency awareness” assessments examined whether participants had correctly formed CS−US associations (see Supplementary Materials). Associations between symptom dimensions, contingency awareness scores and skin conductance responses were investigated using Pearson’s correlations.

### fMRI acquisition and analysis

High resolution structural (T1-weighted) images and blood oxygenation level-dependent (BOLD, T2*-weighted) functional images were acquired and preprocessing procedures applied (see Supplementary Materials). First-level analyses included regressors of interest (acquisition: context, CS+ , CS− and shock; extinction: context, CS+ E, CS−; recall: context, CS+ E, CS+ U, CS−), temporal derivatives, six motion regressors, and regressors to censor outlying volumes.

Region of interest (ROI) analyses were conducted on anatomical amygdala (Harvard-Oxford atlas) and Bed Nucleus Stria Terminalis (BNST; as used in prior research [42]) masks, and a set of a priori regions of threat-based neural circuitry defined as spheres (5 mm radius) around peak activations reported in a meta-analysis of human fear conditioning [vmPFC, subgenual ACC (sgACC), dorsal ACC (dACC), left/right anterior insula, hippocampus [9]]. Although the meta-analysis did not detect significant amygdala activation, noting extensive previous literature on the role of the amygdala and more recent findings identifying a role for the BNST in anxiety-related processing [43], we additionally included bilateral amygdala and BNST ROIs. These ROIs were anatomically defined in order to maximize consistency with previous studies. Power calculations based on 150 participants estimated 80% power to detect effect sizes in ROI data greater than or equal to *r* = 0.23 (power simulations conducted in Mplus).

A series of linear regression analyses determined the statistical significance of activation in each ROI during each phase of fear conditioning (using a Bonferroni-corrected *p* < 0.005). We conducted multi-level analyses to examine unique associations between symptom dimensions and activation of threat circuitry, using the lme4 package (Bates et al., 2014) in R (R Core Team, 2020). Models used a two-level hierarchical data structure, with ROI data nested within each participant’s data in order to account for between-ROI variance. Analyses were conducted for each phase of fear conditioning using commonly used contrasts in prior fear conditioning literature [9, 19, 44]. For acquisition “CS+ vs. CS−” (all trials), for extinction “late CS+ E vs. late CS−” (last four trials of each type), and for recall “early CS+ E vs. early CS+ U” (first four trials of each type). For each phase, one multilevel analysis was conducted in which symptom dimensions were entered as predictor variables. Additional covariates of no theoretical interest were included to account for effects of study site, participant sex, current psychotropic medication use (yes/no), trial order (two task versions with pseudo-randomized trial order) and, for recall only, days between scanning sessions. By conducting a single model for each conditioning phase, we simultaneously control the false positive rate across multiple ROIs and examine unique effects of each symptom dimension. We also conduced whole brain analyses for each task phase and used a permutation-based thresholding procedure with 10,000 permutations [FSL “randomise”; 45].

## Results

### Symptom assessment and factor analysis

Confirmatory factor analyses demonstrated good fit of the tri-level model, consistent with findings from prior work [see Supplementary Materials; 39]. Factor score estimates from this model were saved to generate one score per factor per participant, where values reflect higher levels of General Distress, Fears, and Anhedonia-apprehension, respectively.

### Manipulation checks: contingency awareness and skin conductance response

#### Contingency awareness

**Fig. 1:**
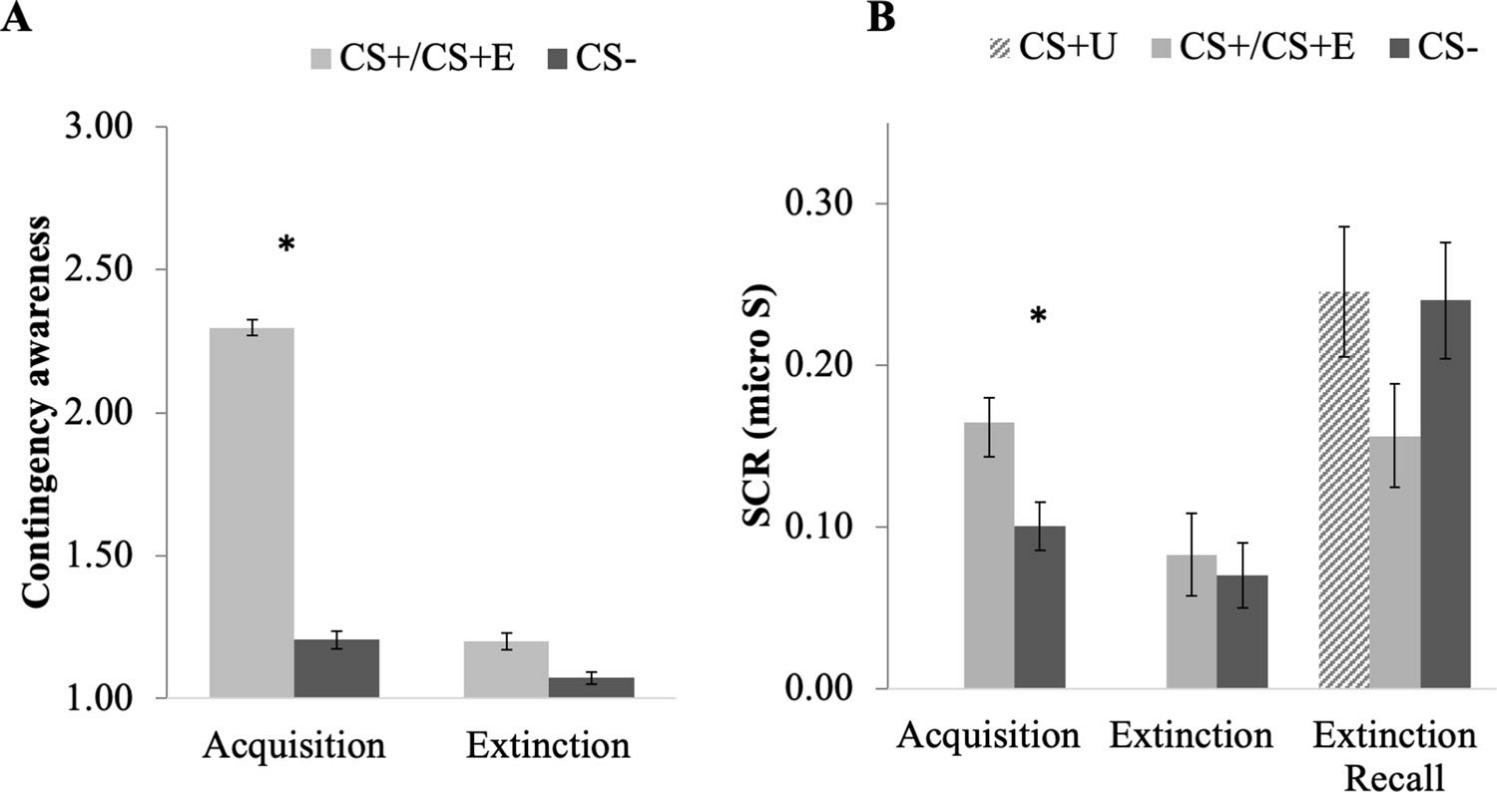
Self-report and skin conductance responses to CS+ and CS− across phases of fear conditioning. **A** Contingency awareness self-report demonstrated significantly greater shock expectancy after CS+ compared to CS− at end of acquisition. There were no significant differences at the end of extinction. **B** Skin conductance showed a similar pattern of effects, with significant differences between CS+ and CS− during acquisition, and no significant differences during extinction and extinction recall. CS+ E = extinguished CS+ (relevant to extinction and recall only); CS+ U = unextinguished CS+ (relevant to recall only), *p < 0.05, error bars represent mean + /− standard error.

There was a significant difference in self-reported contingency awareness at the end of acquisition, adjusting for site, gender, and trial order (comparing averaged CS+ to CS− stimuli, *F*(1, 225) = 80.61, *p* < 0.001, η^2^ = 0.26). Participants rated a higher likelihood of receiving a shock following CS+ compared to CS− (see Fig. 1A). There was no significant difference in contingency awareness between CS+ E and CS− at the end of extinction (*F*(1, 192) = 1.57, *p* = 0.21, η^2^ = 0.01). Participants successfully learned to acquire and extinguish fear associations, as expected. There were no significant associations between contingency awareness and symptom dimensions of General Distress, Fears, or Anhedonia-apprehensions (see Table S2).

#### Skin conductance response

During acquisition, participants demonstrated significantly greater SCR to CS+ compared to CS−, adjusting for site, gender, and trial order (comparing averaged CS+ to CS−, *F*(1, 182) = 8.93, *p* = 0.003, η^2^ = 0.05; Fig. 1B). During extinction, SCR was significantly higher to the first four CS+ E vs. first four CS− trials (*F*(1, 152) = 11.11, *p* = 0.001, η^2^ = 0.068) and there was a significant reduction in SCR between the first four and last four CS+ E trials (*F*(1, 152) = 20.11, *p* < 0.001, η^2^ = 0.118). There was no significant difference in SCR between the last four CS+ E and four CS− extinction trials (*F*(1, 152) = 0.36, *p* = 0.55, η^2^ = 0.002). During recall, there was no significant difference in SCR between CS+ U and CS+ E during the first four trials (*F*(1, 130) = 1.27, *p* = 0.26, η^2^ = 0.01; see Fig. 1B). These results show that participants acquired conditional fear, indicated by higher arousal responses to CS+ than CS− during acquisition, and then extinguished their fear by the end of extinction. Unexpectedly, SCR to extinguished and unextinguished cues did not significantly differ at extinction recall. There were no significant associations between skin conductance responses and symptom dimensions of General Distress, Fears, or Anhedonia-apprehension.

### Threat neural circuitry and symptoms of anxiety and depression

**Fig. 2:**
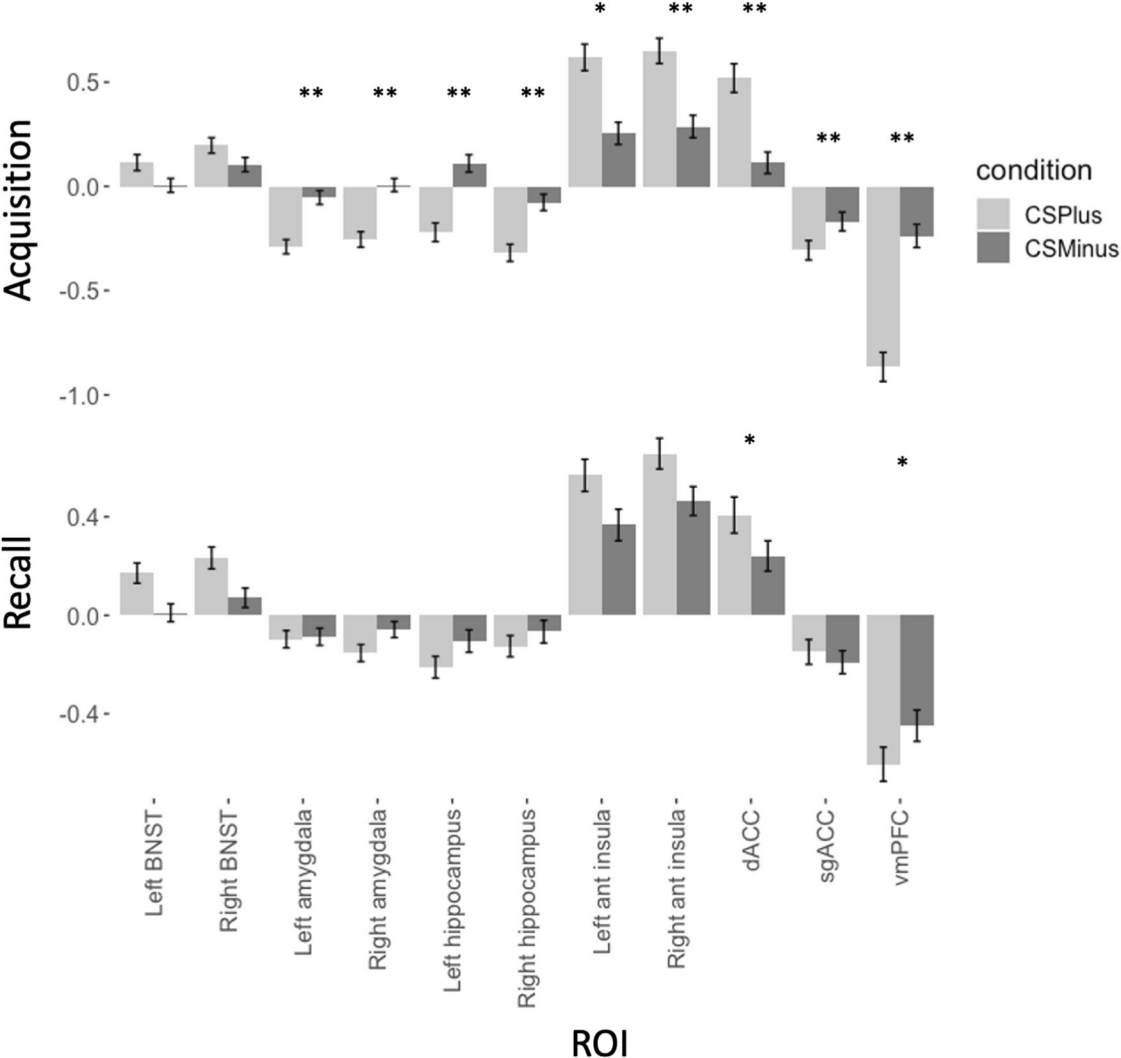
Parameter estimates for activation across key ROIs of threat neural circuitry during acquisition and recall, separated by CS−type. During acquisition, significant differences in activation to CS+ and CS− were observed across all ROIs except left amygdala. During early recall, differences were observed in right anterior insula, dorsal ACC, and vmPFC. CS+ E = extinguished CS+ (relevant to extinction and recall only); CS+ U = unextinguished CS+ (relevant to recall only), *p < 0.005; **p < 0.001.

Multilevel ROI analyses examined main effects of stimulus type (significant differences in neural reactivity to CS’s across all individuals) and associations with symptom dimensions (Table 2). During acquisition there was no significant main effect of stimulus type on activation in threat neural circuitry, and no significant effect of General Distress, Fears, or Anhedonia-apprehension. Post-hoc tests of the main effect demonstrated significantly greater activation to CS+ than CS− in the right and left BNST, insula and dorsal ACC, and greater deactivation to CS+ than CS− in right and left amygdala, hippocampus, subgenual ACC, and vmPFC (Fig. 2, Table S3; see Supplementary Materials for further details on amygdala effects).

**Table 2:** Results from multilevel analyses examining the differential activation of threat neural circuitry ROIs across phases of fear conditioning and associations with symptom dimensions of general distress, fears and anhedonia-apprehension.

	Parameter estimate	Confidence interval	*p* value
Fear acquisition (all CS+ vs. all CS−)			
(Intercept)	0.11	−0.04 0.26	0.143
General Distress	0.00	−0.06, 0.07	0.947
Fears	−0.05	−0.12, 0.01	0.121
Anhedonia-apprehension	0.04	−0.03, 0.10	0.244
Fear extinction (late CS+ vs. late CS−)			
(Intercept)	−0.02	−0.17, 0.14	0.831
General Distress	−0.01	−0.08, 0.06	0.771
Fears	−0.02	−0.10, 0.06	0.576
Anhedonia-apprehension	0.13	0.06, 0.20	0.001*
Extinction recall (early CS+ U vs. early CS+ *E)*			
(Intercept)	0.16	0.02, 0.30	0.030*
General Distress	0.01	−0.05, 0.08	0.659
Fears	−0.06	−0.13, 0.01	0.103
Anhedonia-apprehension	0.02	−0.04, 0.09	0.491

*p < 0.05.

**Fig. 3:**
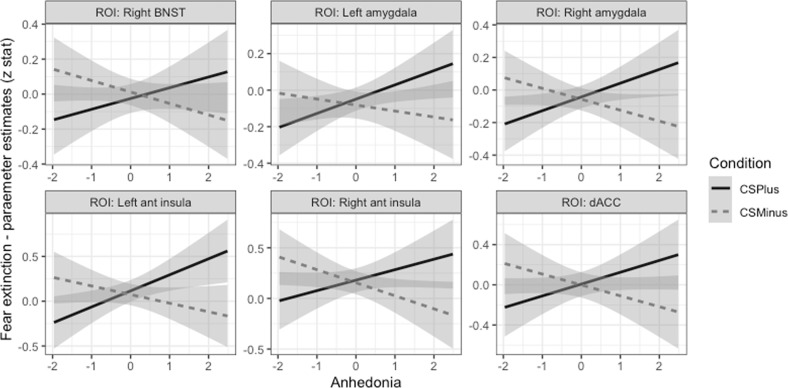
Plots demonstrating patterns of associations between neural activation and the symptom dimension of Anhedonia-apprehension during late extinction. High levels of Anhedonia-apprehension were associated with greater activation to CS+ (the CS undergoing extinction) than CS− across all significant ROIs.

During late extinction, there was no main effect of stimulus type on reactivity of threat neural circuitry ROIs, and no significant effects in post-hoc ROI tests. There was a significant interaction effect of Anhedonia-apprehension by stimulus type on threat circuitry activation, with post-hoc tests demonstrating that higher Anhedonia-apprehension was associated with greater reactivity to CS+ E and reduced reactivity to CS− in right BNST, bilateral amygdala, bilateral anterior insula, and dorsal ACC (see Fig. 3).

During early recall, there was a significant main effect of stimulus type on threat circuitry ROI activation, but no significant effects of the symptom dimensions. Post-hoc tests of the main effect demonstrated greater activation to CS+ E than CS+ U in the dACC and vmPFC (Fig. 2).

Participant sex, psychotropic medication use, study site, task version, and mean days between scans (recall only) were included as a covariate in all multilevel analyses. No significant effects of any of these covariates were observed.

Whole brain analyses demonstrated that during acquisition, significant main effects of stimulus type were observed in the ACC, insula, dorsomedial/lateral PFC occipital cortex and cerebellum (CS+ > CS−) and in a large distributed cluster encompassing vmPFC, amygdala, and hippocampus (CS− > CS+ ; Table S4). Symptoms of Anhedonia-apprehension were associated with activation in clusters located in the occipital and posterior cingulate cortices (see Table S5). During late extinction, there were no suprathreshold clusters for the main effect of stimulus type, but symptoms of Anhedonia-apprehension were associated with activation in a spatially distributed cluster, encompassing the insula, anterior cingulate cortex, occipital cortex, and dorsal/ventral lateral regions of PFC, amygdala and hippocampus (Table S3). During early recall, there were no suprathreshold clusters for the main effect of stimulus type and no significant associations with symptom dimensions.

## Discussion

Using a dimensional model of anxiety and depression, we did not find evidence of an association between threat neural circuitry during a Pavlovian fear learning paradigm and symptom dimensions of Fears or General Distress. In contrast to predictions, we observed an association between Anhedonia-apprehension and elevated reactivity to an extinguished threat stimulus (CS+ E) in amygdala, anterior insula and ACC during late extinction. These surprising findings suggest that activation of “threat neural circuitry” may be associated with variance in a symptom dimension related to positive affect (Anhedonia-apprehension). We failed to find evidence for an association with a symptom dimension representing variance shared across anxiety disorders (i.e., Fears) or a symptom dimension representing shared features of anxiety and depression (General Distress). Anhedonia-apprehension may play a previously underrecognized role in neurobiological learning processes thought to be critical in the development, maintenance, and treatment of anxiety disorders.

### Relationships between symptom dimensions and activation of “threat neural circuitry”

Previous studies suggested associations between symptoms, or diagnoses, of anxiety disorders and altered functioning of threat neural circuitry. However, prior work lacks consensus regarding the specific brain regions, task phases, and anxiety measures involved. The current study, which we believe to be the largest to date, examined activation in key regions of threat neural circuitry across three phases of fear conditioning (acquisition, extinction, and recall), using a dimensional model of anxiety and depression symptoms. Despite evidence of activation of threat neural circuitry during fear learning at the group-level, we did not observe the expected individual differences associations between Fears or General Distress and activation of this neural circuitry during any phase of fear conditioning. These findings align somewhat with theoretical advances in the neuroscience of anxiety, which propose a differentiation of neural circuitries that support behavioral and physiological responses to fear from conscious states of fear or anxiety [43]. Our findings show that both cortical and subcortical brain regions are consistently recruited when differentiating threatening from non-threatening stimuli during a fear learning task, but that the degree of activation across these circuitries was not significantly associated with trait level anxiety or emotional distress. Individual differences in these traits may be better represented by functioning of higher order brain regions (although this was not detected in exploratory whole brain analyses) or, for example, by the interaction and functional connectivity between cortical and subcortical regions implicated in threat and fear/anxiety processes.

The absence of expected findings should be considered in light of some caveats. First, although the study was well-powered to detect small effects (*r* > 0.23), it may be that very small effects are present but did not meet the threshold for statistical significance (fMRI has characteristically small effect sizes due to the low signal-to-noise ratio [46]). Second, although ~34% of our sample met criteria for a current anxiety or depressive disorder, we cannot rule out the possibility that effects might have been different in a more severely anxious or depressed clinical sample. Finally, although the majority of prior work in this area has focused on activation of neural regions, it may be that functional connectivity between regions is a more accurate reflection of network functioning and may be more relevant to understanding altered neural processing in psychopathology.

Unexpectedly, we observed an association between symptoms of Anhedonia-apprehension and activation of amygdala, anterior insula and dACC during late extinction. Individuals with high levels of Anhedonia-apprehension (or low positive affect) showed greater activation to CS+ E than CS−, whereas those with low levels of Anhedonia-apprehension showed the opposite pattern of effects, with deactivation to the CS+ E in most of these regions. These regions are all associated with threat-reactivity, rather than inhibition or memory-related threat processing, however, exploratory whole brain analyses detected additional effects in lateral prefrontal cortex during late extinction. These findings suggest that higher levels of Anhedonia-apprehension may be associated with greater levels of threat responding during extinction, and may also impact higher-order processing that may contribute to the conscious experience of anxiety or fear.

The symptom dimension of Anhedonia-apprehension is so named due to the loading of items related to both low positive affect and apprehension, but low positive affect is the predominant feature. Low positive affect is strongly associated with depression but is also characteristic of some anxiety disorders [47, 48]. Anhedonic low positive affect is related to reward processing, including the anticipation of future reward, experience of reward “in-the-moment” (reward consumption), and reward learning, among others [28, 49]. Aspects of fear extinction tap into reward processing; in particular, absence of the US during extinction trials (i.e. the ‘relief’ when an anticipated shock is not received) is connected with reward processing [50, 51]. As symptoms of anhedonia have been associated with deficits in reward-learning processes [52, 53], it is plausible that the associations we observed represent deficits in “relief-reward” learning, thus accounting for continued elevated threat reactivity to the CS+ during extinction. Alternatively, depression has been previously associated with impairments in updating associations when contingencies change [54, 55]. Conceivably, symptoms of Anhedonia-apprehension are associated with impaired cognitive flexibility and reduced safety learning of a previously threatening stimulus, manifesting in elevated threat reactivity to CS+ during extinction. Future work exploring the effects of Anhedonia-apprehension on altered functioning of this circuitry that directly contrasts threat-based and reward-based learning may explore these possibilities. As a novel, unexpected finding, it is particularly important that this effect also be examined for replicability in future work.

### Engagement of “threat neural circuitry” across phases of fear conditioning

During fear acquisition, we observed a pattern of threat circuitry activation consistent with recent meta-analytic findings (CS+ > CS− in dACC, anterior insula, CS− > CS+ in hippocampus, sgACC, and vmPFC [9]), with the exception of the amygdala. Although the amygdala is demonstrated as central to fear learning in animal studies, lack of significant findings from human neuroimaging have been attributed to reduced threat salience in human studies, or an inability of fMRI to detect transient amygdala responses [9, 56, 57]. Other work has suggested a more prominent role for amygdala activation during the presentation of an aversive stimulus (e.g., US shock) rather than during the anticipation (i.e., CS+) of this stimulus [58]. We instead show deactivation of the amygdala to CS+ during acquisition. While the functional role of deactivation of the amygdala remains unclear, discriminatory functioning of the amygdala in response to threatening vs. non-threatening cues is consistent with the broader literature. Other fear conditioning studies have also demonstrated amygdala deactivation in adolescents and young adults [59, 60]. In addition, deactivation findings may have been masked in other work that reported neither CS− > CS+ contrasts nor separate effect size estimates for each CS [9, 61]. Further examination of this effect, including more detailed analysis of the functional roles of discrete amygdala sub-nuclei [43], is required to better understand the functional role of amygdala deactivation and potential developmental differences in amygdala functioning.

During late extinction, we observed no significant differences in threat circuitry activation in ROI or whole brain analyses. A meta-analysis examining the entire phase of extinction (not just late extinction) demonstrated activation of anterior insula and dACC to CS+ E vs. CS− [44]. We focused on late extinction, as that is the point by which participants normally learn that the CS+ E is no longer a predictor of threat (and thus significant threat circuitry activation would not be expected) and hence is the point at which deficits as a function of anxiety are most likely to be observed. Indeed, it is the phase in which we observed differences in neural activation across key regions of threat circuitry as a function of Anhedonia-apprehension.

During early recall, we observed significant differences in responding to CS+ U vs. CS+ E in dACC and vmPFC. These findings differ from those reported in a meta-analysis, which identified limited activation of vmPFC, dmPFC, and hippocampus during extinction recall [44]. However, the sensitivity and reliability of findings from meta-analyses are limited by differences in methodologies and analytic procedures of studies included. Our findings represent the largest single study of human fear conditioning with fMRI to date, which likely resulted in greater power to detect effects. Although we observed differences in neural responding to CS+ U and CS+ E, there were no significant differences in skin conductance response during extinction recall, and skin conductance responses to all stimuli were higher during recall compared to acquisition and extinction. Elevated skin conductance responses during recall may indicate contextual renewal of fear responses caused by reintroduction of the CS+ U that was not presented during extinction, which may have signaled a change back to the acquisition context [62–64].

### Limitations

Participants in this study were aged 18–19 years at the time of testing due to their involvement in a larger, longitudinal project examining developmental changes in threat and reward functioning from adolescence to early adulthood. This narrow age range limits the generalizability of the current cross-sectional findings. In particular, although adolescence represents a period of increased risk for onset of anxiety and depression, many individuals who ultimately experience these conditions have not yet done so by late adolescence. In addition, adolescence and early adulthood represent a period of brain maturation, particularly in prefrontal cortical regions, some of which were investigated here. Therefore, limited variance observed in relation to symptom dimensions may be in part because individual differences in threat neural circuitry functioning have not yet reached their final mature state, or have not yet manifested as symptoms in the age group studied. Future work might investigate the consistency of these associations across time in longitudinal studies, in participants with a broader age range and in participants with more severe symptoms to assess how sustained and generalizable effects are.

## Supplementary information

Supplemental materials

## Footnotes

1 Others may prefer to use the term “bifactor” whereas we prefer the term hierarchical as it does not imply the number of factors on which most indicators load, whereas the term bifactor originated in IQ research to describe a model in which all indicators loaded on two factors (a general factor and a specific factor)
