# Supplementary material for: Dysregulation of threat neurocircuitry during fear extinction: the role of anhedonia
Source: Neuropsychopharmacology. 2021 Apr 8;46(9):1650–7. doi: 10.1038/s41386-021-01003-8 (PMC8280223; doi:10.1038/s41386-021-01003-8)
Supplement: Supplementary file 1 — Supplemental materials [file 41386_2021_1003_MOESM1_ESM.docx]

Supplemental materials

### Participants

Participants were recruited from university and college campuses, community centers and community notice boards in Los Angeles and Chicago. Recruitment occurred as part of the larger ‘Brain, Motivation and Personality Development’ (BrainMAPD) study, a multi-site longitudinal project investigating positive and negative valence functioning in late adolescence to early adulthood, conducted at the University of California, Los Angeles and Northwestern University (R01 MH100117). Participants were recruited based on their scores on self-reported trait Neuroticism using the Eysenck Personality Questionnaire-Neuroticism, EPQ-N^25^ and Reward Sensitivity using the Behavioral Activation Scale, BAS^26^ from among a total of 2,461 individuals who completed screening. Participants were recruited to ensure sampling from high/mid/low ranges (tertiles) on both scales, with oversampling from the two diagonals of the bivariate space defined by the quasi-orthogonal EPQ-N and BAS scales (i.e., high EPQ-N/high BAS, low EPQ-N/low BAS, mid EPQ-N/mid BAS, high EPQ-N/low BAS and low EPQ-N/high BAS). This approach aimed to maximize variance in threat- and reward-related sensitivity within our sample to ensure diversity in symptom profiles.

Inclusion criteria were: aged 18-19 years old (at the time of screening), right-handed (assessed using the Edinburgh handedness inventory) and fluent in English. Exclusion criteria were: (1) traumatic brain injury with evidence of neurological deficits, neurological disorders, or severe or unstable medical conditions that might be compromised by or interfere with study participation (assessed using Ohio State University Traumatic Brain Injury Identification Method^27^), (2) any condition that interferes with acquisition/interpretation of fMRI data (e.g., severe claustrophobia, central nervous system illness, nonremovable metal in the body), (3) pregnancy, (4) color blindness (given the need to differentiate between colored CS stimuli, assessed using the Isihara Test), (5) lack of right-handed dominance (assessed using the Edinburgh Handedness inventory^28^), (6) lifetime psychotic symptoms, (7) lifetime bipolar I disorder, and (8) clinically significant substance or alcohol use disorder in the past 6 months^29^, and (9) antipsychotic medication usage (6-9 assessed using the Structured Clinical Interview for DSM-5^30^).

A total of 272 participants recruited into the study completed the current task (182 female, mean age = 19.16 years, SD = 0.52). A number of participants were excluded from analyses for various reasons (e.g., excessive motion, reported falling asleep, technical issues, see Table S1). In final analyses, n=229 had usable data for fear acquisition, n=220 for fear extinction and n=212 for extinction recall; 260 unique individuals overall). The racial/ethnic composition of the final sample (*n* = 260) was as follows: White (*n* = 141, 88.1%), Black or African American (*n* = 21, 8.1%), Asian (*n* = 73, 45.6%), American Indian or Alaska Native (*n* = 4, 1.5%), Multiracial (*n* = 20, 7.7%), none by choice (*n* = 1, 0.4%), Hispanic or Latino (*n* = 68, 26.2%) and not Hispanic or Latino (*n* = 192, 73.8%).

Table S1. Reasons for participant exclusion across task phases.

|  | Acquisition | Extinction | Recall |
| --- | --- | --- | --- |
| Total n included | 229 | 220 | 212 |
| Reasons for exclusion: | | | |
| Excessive motion^#^ | 38 | 41 | 39 |
| Reported falling asleep during scan | 4 | 8 | 0 |
| Technical failure* | 2 | 4 | 4 |
| Had reported being asleep for acquisition | n/a | n/a | 7 |
| Less than 48 hours or more than 14 days between scans | n/a | n/a | 5 |
| Did not complete scan | n/a | n/a | 6 |

^#^Defined as >10% outlier scans, see ‘fMRI analysis’ section, *failure in synchronizing experimental task with scan onset

### Trilevel model

Confirmatory factor analyses (CFA) demonstrated a good fit of the tri-level symptom model^23,24^ with self-reported symptom data in the current sample provided at the time of the MRI session. Factor score estimates from this model were saved and used to represent the trilevel model symptom dimensions of general distress, fears and anhedonia-apprehension. The three factor scores are quasi-orthogonal: correlations of the general distress factor scores with the fears and anhedonia-apprehension factor scores equaled .09 (*p* = .16) and -.09 (*p* = .13), respectively. Similarly, the fears and anhedonia-apprehension factor scores correlated .06 (*p* = .29). Consequently, associations with each dimension’s factor scores can be considered unique of the others.

### fMRI task

Participants completed a differential Pavlovian Fear Learning Task widely used in prior studies of healthy and anxious participants^6,39^. This slow event-related fMRI paradigm consisted of four phases: habituation, acquisition, extinction (all conducted on day 1) and recall (conducted on day 2, 1-7 days later). During habituation, participants viewed each of three CS images for four 6-second trials, to reduce novelty. During acquisition, participants viewed images of two CS+ stimuli and one CS- stimulus. Images were office or conference rooms (context) with different colored lights (red/yellow/blue) as CS stimuli (color order and context images were counterbalanced across participants). During each trial, participants first viewed the context image (3sec), followed by the CS embedded in the context (6sec). There were 8 trials of each CS+ (16 trials total) and 16 trials of the CS-. Five (out of eight) of the CS+ trials of each type were followed immediately by a mild electric shock applied to the left bicep. In acquisition analyses, responses to the two CS+ stimuli were combined. During extinction, participants viewed 16 trials of one CS+ (the ‘extinguished’ CS+ now termed the CS+E) and 16 trials of the CS-, none of which were followed by shock. During recall, participants viewed 8 trials of the CS+E, 8 trials of the CS+ that was not presented during extinction (the ‘unextinguished’ CS+ now termed the CS+U) and 16 trials of the CS-. During all task phases, inter-trial intervals varied from 12-18sec (mean 15sec) and included a jitter of 125ms per trial to reduce slice timing bias. The task was programmed in E-Prime (version 2.0 SP1) and presented to participants using a mirror and projector system.

Shocks consisted of 10 pulses of 1ms pulse duration, delivered at 20Hz frequency (total duration = 500ms). Shocks were delivered using a DS7a constant current high voltage stimulator (Digitimer Ltd, England) at UCLA and a STMISOC constant voltage stimulator (Biopac Systems Inc, USA) at Northwestern. Shock levels were determined during a ‘work-up’ procedure conducted on Day 1 before scanning. In this procedure, participants were presented with shocks of increasing intensity and were asked to rate each on a pain scale of 1-10 (1 = ‘not at all painful’, 10 = ‘most pain imaginable’). Participants were informed we aimed to reach a level of shock that was ‘uncomfortable but not painful’ and ‘took some effort to tolerate’ (i.e., a rating of 5-6 that they were willing to tolerate for the experiment).

At the end of each task phase, participants were asked contingency awareness questions. For each of the CS stimuli, participants rated the ‘likelihood of receiving a shock if you saw this image again’, on a 3-point scale, 1 ‘high’, 2 ‘moderate’, 3 ‘low’. These responses were reverse-coded during analysis so that higher scores indicated greater likelihood.

### Skin conductance responses (SCR) acquisition and analysis

Galvanic skin conductance was recorded throughout all task phases using a GSR100c amplifier (Biopac Systems Inc., USA) and was digitized using AcqKnowledge Data Acquisition and Analysis Software (Biopac Systems Inc., USA). Data were sampled at a rate of 1kHz, with a gain of 5 μS/V and further processed using the software ANSLAB^40^. A total of 272 participants completed the task. Data were visually inspected, movement artifacts were edited out (on a trial-by-trial basis) and data that still had poor quality signal following this step (i.e., technical issues with data collection, lack of variance in acquired data or excessive motion artifacts that could not be edited out; n=54) were removed, leaving *n* = 218 with usable skin conductance data. Skin conductance responses (SCR) to the CS were calculated by subtracting pre-CS baseline skin conductance level (SCL; -2 to 0s before CS onset) from the maximum CS SCL (occurring between 0 to 6s after CS onset). Data were normalized using the natural logarithm of 2+SCR. As described below, 43 of the original 272 participants were excluded from fMRI analyses due to motion or technical issues. In total, 184 participants had usable fMRI data and usable skin conductance data. A comparison of participants with usable skin conductance data with (n=184) and without (n=34) usable fMRI data demonstrated no significant differences in skin conductance responses to CS+ and CS- stimuli (*p* > .32).

### fMRI data acquisition

Data were acquired on Prisma 3.0 Tesla whole-body scanners using 64-channel head coils (Siemens Medical Systems, Iselin, New Jersey) at the UCLA Ahmanson-Lovelace Brain Mapping Center and the Northwestern University Center for Translational Imaging. High resolution structural images (T1-weighted) were acquired using a magnetized prepared rapid acquisition gradient echo (MPRAGE) sequence using 0.8mm isotropic voxels, TR/TE/flip angle=2300ms/2.99ms/7°, FOV= 256mm^2^, 208 slices. Blood oxygenation level-dependent (BOLD, T2*-weighted) functional images were acquired parallel to the AC-PC line using Siemens AutoAlign function, using 2mm isotropic voxels, TR/TE/flip angle=2000ms/25ms/80°, FOV = 208mm^2^, 64 slices, multiband acceleration factor=2, sequential slice acquisition, 380 volumes (per task phase).

### fMRI analysis

Raw dicom files were converted to NIFTI format using dcm2nii (MRIcroN, <http://www.cabiatl.com/mricro/mricron/dcm2nii.html>). Data were processed and analyzed using FSL (FMRIB’s Software Library, [www.fmrib.ox.ac.uk/fsl](http://www.fmrib.ox.ac.uk/fsl)). Structural data was corrected for spatial intensity variations (bias field correction) using FAST (FMRIB’s Automated Segmentation Tool^41^) and brain extraction was performed using optiBET (optimized brain extraction^42^).

Functional data was first assessed for outlier volumes (75^th^ percentile +1.5 time interquartile range) based on framewise displacement (average of rotation and translation parameter differences, using weighted scaling^43^ as implemented in the fslmotionoutliers function). Runs with >10% outliers were not included in group analyses (n=38, 14%). For included scans, outlier volumes were censored in first level analyses by including a regressor with a single time point corresponding to each outlying volume. Functional data were brain extracted using BET (Brain Extraction Tool, FSL^44^) and bias field corrected using N4BiasFieldCorrection, run twice (ANTS registration suite^45^).

fMRI data processing was carried out using FEAT (FMRI Expert Analysis Tool) Version 6.00. Registration to high resolution structural space images was carried out using FLIRT^46,47^. Registration from high resolution structural to standard space was then further refined using FNIRT (nonlinear registration)^48,49^. The following pre-statistics processing was applied: motion correction using MCFLIRT^47^, slice-timing correction using Fourier-space time-series phase-shifting, spatial smoothing using a Gaussian kernel of FWHM 4.0mm, grand-mean intensity normalisation of the entire 4D dataset by a single multiplicative factor, and high-pass temporal filtering (0.01Hz) to remove low frequency artifacts.

First-level analyses of neural activation included regressors of interest (context, CS+, CS- and shock) and temporal derivatives, six motion regressors and additional regressors to censor outlying volumes (described above). Time-series statistical analysis was carried out using FILM with local autocorrelation correction^50^. Contrasts were computed as follows: i) for acquisition, CS+ > CS- and CS- > CS+, ii) for extinction, late CS+E > late CS- and late CS- > CS+E; iii) for extinction recall, early CS+U > early CS+E and early CS+E > early CS+U.

Second level whole brain analyses were conducted by running a single generalized linear model. Regressors included were: symptom dimensions of general distress, fears and anhedonia-apprehension (see ‘Symptom Assessment’ above), site, gender and task version (which of the counter-balanced versions of the task each participant received). Analyses for extinction recall additionally included a mean-centered regressor for the ‘number of days between scans (scan 1 = acquisition and extinction, scan 2 = extinction recall). Estimates of associations between symptom dimensions and brain activation were therefore unique to the regressor of interest, controlling for variance associated with other regressors. Additionally, as factor scores for each symptom dimension were quasi-orthogonal, results associated with one symptom dimension can be considered to be independent of the other symptom dimensions. Resulting Z (Gaussianised T/F) statistic images were thresholded using a permutation-based procedure with 10,000 permutations (FSL ‘randomise’; 33).


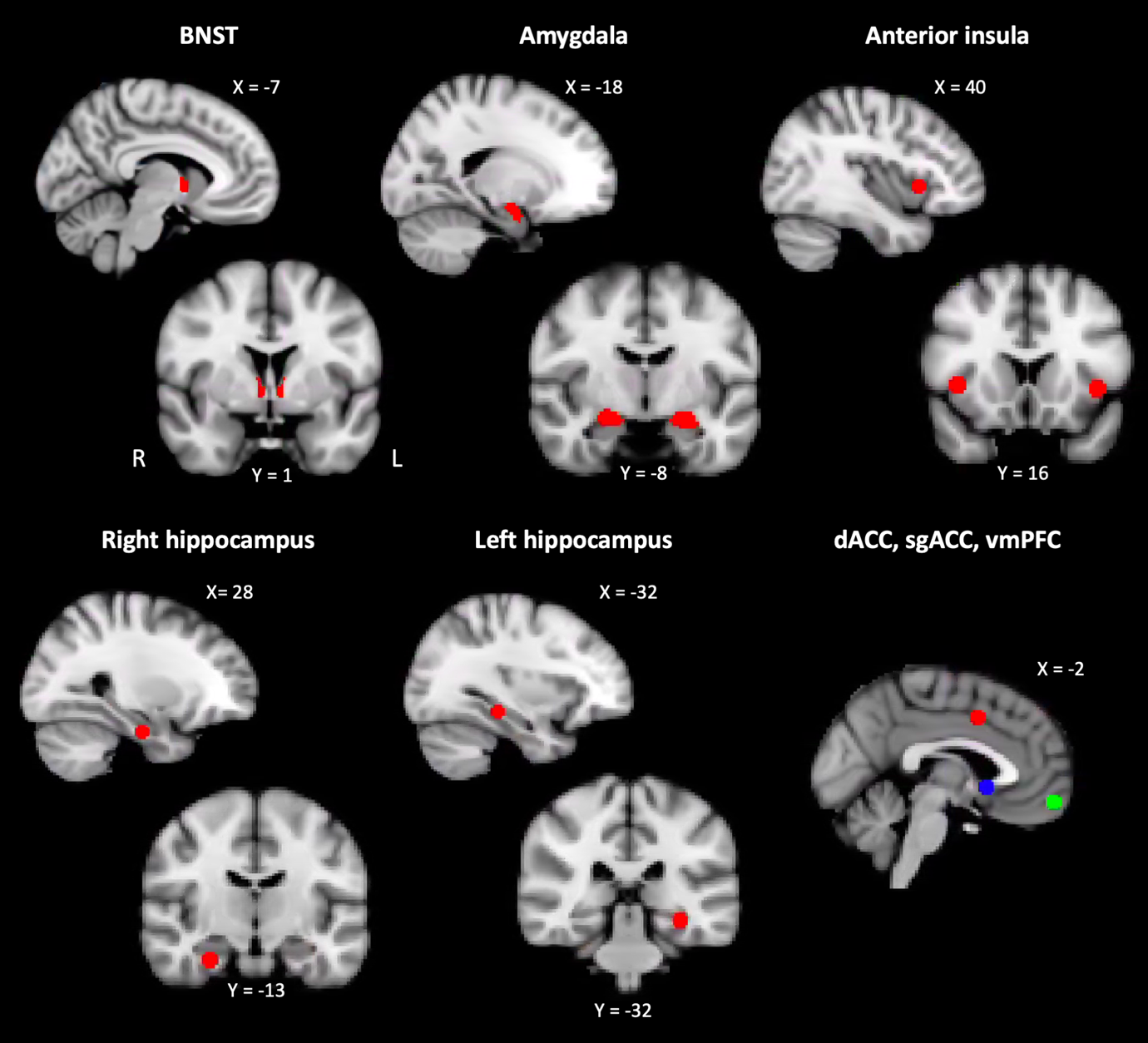


**Figure S1.** ROIs superimposed on standardized MNI 152 anatomical image. X and Y values indicate MNI coordinates of slices presented. dACC = red, vmPFC = green, sgACC = blue. BNST and amygdala ROIs were anatomically defined. The remaining ROIs were 5mm spheres around functional peaks reported in a meta-analysis of human fear conditioning [9].

**Supplemental results**

Table S2. Pearson’s correlation coefficients between symptom dimensions, contingency awareness and skin conductance responses.

|  | General Distress | Fears | Anhedonia-apprehension |
| --- | --- | --- | --- |
| ***Symptom dimensions (n=260)*** | |  |  |
| General Distress | 1.00 | 0.10 | 0.10 |
| Fears | 0.10 | 1.00 | -0.06 |
| Anhedonia-apprehension | 0.10 | -0.06 | 1.00 |
| ***Contingency (n=260)*** | |  |  |
| Acquisition: CS+ | -0.07 | 0.03 | -0.02 |
| Acquisition: CS- | -0.09 | -0.08 | -0.06 |
| Extinction: CS+ | -0.11 | -0.02 | -0.05 |
| Extinction: CS- | -0.02 | -0.07 | -0.11 |
| ***Skin conductance response (n=208)*** | | |  |
| Acquisition: CS+ | 0.03 | -0.11 | -0.11 |
| Acquisition: CS- | 0.06 | -0.08 | -0.10 |
| Extinction: CS+E | 0.01 | 0.02 | -0.02 |
| Extinction: CS- | -0.06 | -0.06 | 0.02 |
| Recall: CS+E | -0.06 | -0.06 | 0.04 |
| Recall: CS+U | 0.00 | -0.02 | 0.09 |

Table S3. Main effects of contrast across regions of interest for each phase of fear conditioning

|  | Acquisition  (CS+ > CS-) | | Extinction  (Late CS+E > Late CS-) | | Recall (Early CS+E > Early CS+U) | |
| --- | --- | --- | --- | --- | --- | --- |
|  | coeff | p-val | coeff | p-val | coeff | p-val |
| Left BNST | 0.10 | 0.287 | -0.06 | 0.508 | 0.14 | 0.087 |
| Right BNST | 0.12 | 0.190 | -0.06 | 0.567 | 0.17 | 0.062 |
| Left amygdala | -0.34* | < .001 | 0.10 | 0206 | -0.06 | 0.402 |
| Right amygdala | -0.37* | < .001 | 0.08 | 0.305 | -0.09 | 0.244 |
| Left hippocampus | -0.47* | < .001 | 0.03 | 0.736 | -0.11 | 0.281 |
| Right hippocampus | -0.33* | < .001 | 0.01 | 0.927 | -0.04 | 0.652 |
| Left insula | 0.40* | .003 | -0.05 | 0.720 | 0.09 | 0.413 |
| Right insula | 0.42* | .001 | -0.08 | 0.518 | 0.19 | 0.078 |
| dACC | 0.79* | < .001 | 0.00 | 0.980 | 0.31* | 0.006 |
| sgACC | -0.40* | < .001 | -0.06 | 0.540 | -0.01 | 0.919 |
| vmPFC | -0.76* | < .001 | 0.04 | 0.798 | -0.25* | 0.034 |

*** denotes significant effect, p < .05**

***Additional amygdala ROI analyses***

Given the unexpected deactivation of bilateral amygdala during CS+ presentation, relative to implicit baseline, we conducted a more detailed examination of amygdala responding to CS stimuli during fear acquisition. We sought to examine two questions. First, as fear acquisition involves learning about cue-threat associations across successive trials, we examined whether the pattern of amygdala responding would vary in early trials (the first four trials of each type before associations are well established), compared to late trials (the last four trials of each type, when learning has likely occurred). For completeness, we also display amygdala responses during the remaining ‘mid’ acquisition trials (8 trials of each type). As shown in Figure S1, there is a similar pattern of responses in both left and right amygdala, with deactivation to CS+ stimuli becoming stronger from early, to mid, to late trials. CS- trials do not deviate much from implicit baseline, with a slight trend toward deactivation by the late phase of acquisition. In comparison, both context images and shock presentation show positive activation of the amygdala. This pattern of effects would suggest an association between stronger threat anticipation (presumed to occur later in acquisition) and stronger deactivation of the amygdala.

Figure S2. Z-score estimates in right and left amygdala ROIs during early, mid and late CS trials of acquisition. For comparison, amygdala responses during context images and shock presentation are displayed. Error bars represent mean +/ SE.

Secondly, given concerns regarding the ability to detect transient amygdala responses with fMRI, we explored the timecourse of amygdala activation across early, mid and late acquisition trials. Given that each CS trial was 6s in duration, we were hypothesised that an initial positive amygdala activation response would be observed, followed by a deflection back to baseline (or below), preventing observation of overall activation when averaged across the total 6s. We extracted parameter estimates of amygdala activation for each 2s TR from early (first four trials), mid (middle 8 trials) and late (last four trials) for each CS type, then computed averaged timecourses across participants (Figure S2). In both right and left amygdala, a prominent deactivation peak emerges over the course of acquisition, peaking around 8s after CS+ onset. We find no evidence to suggest there are transient early responses (occurring within 2s TR time windows) to CS images in the amygdala which are masked by the 6s duration of the CS image.


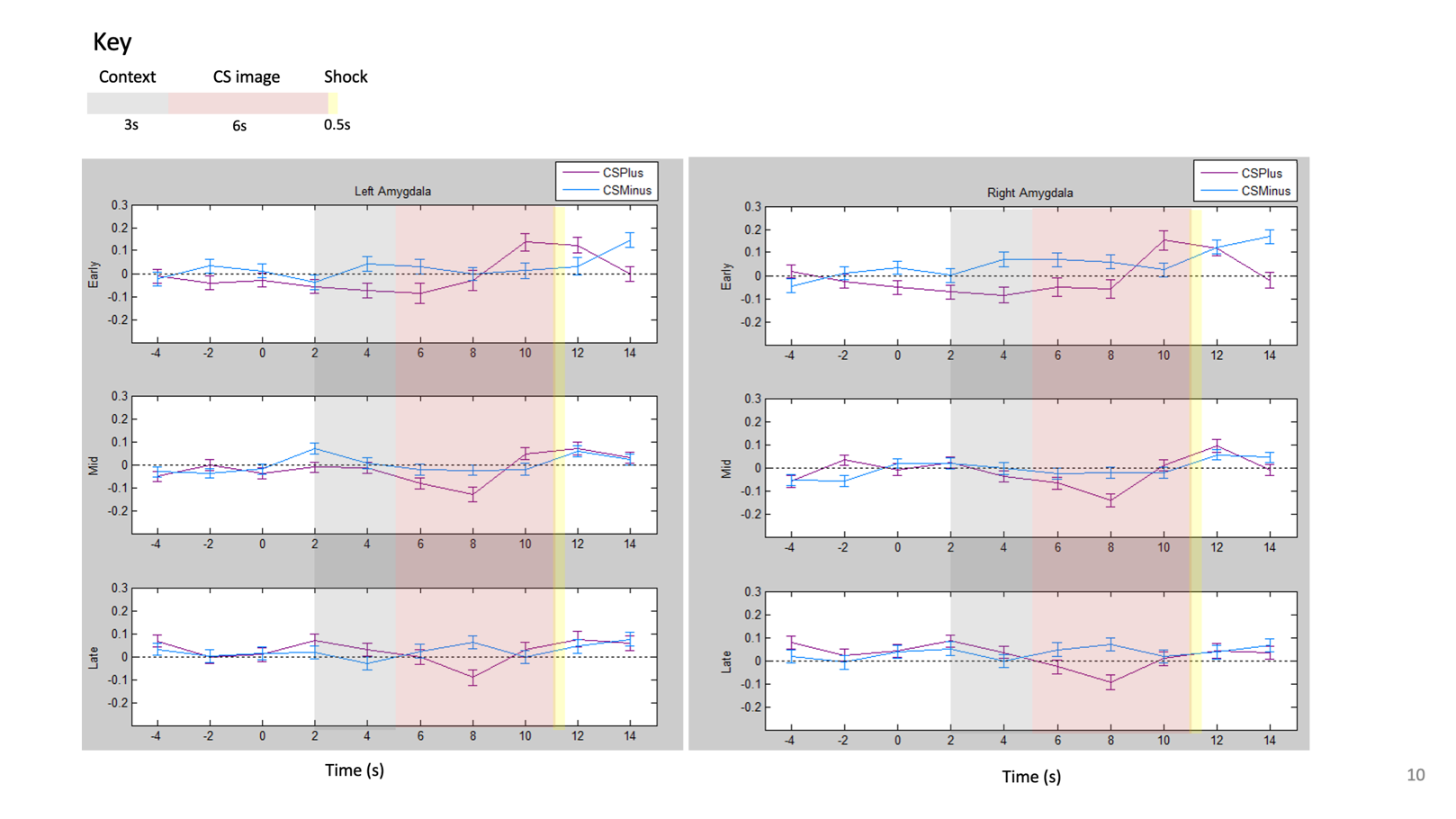


Figure S3. Timecourse of amygdala responses to CS+ and CS- images in the left and right amygdala in early (upper), middle (mid) and late (lower) phases of acquisition. Colored shading indicates the timing of trial elements (offset relative to the timing on the x-axis to approximate a 5-sec delay in HRF peak, with 0s representing the onset of CS image, but red shading indicating the anticipated HRF peak to CS beginning 5s later). Error bars represent mean +/- SE.

**Results: whole brain analyses**

Table S4. Clusters of significant differential activation to CS+ and CS-/CS+E cues across phases of fear conditioning

|  | Voxels | X | Y | Z | | |
| --- | --- | --- | --- | --- | --- | --- |
| **Acquisition** |  |  |  |  | | |
| *CS+ > CS-* |  |  |  |  | | |
| SFG, supplementary motor cortex, paracingulate gyrus, ACC/PCC, L/R caudate, L/R putamen, L/R hippocampus | 4494 | -2 | -6 | 8 | | |
| Insula, IFG, precentral gyrus, orbitofrontal cortex, R putamen | 1361 | 46 | 16 | -2 | | |
| Insula, IFG | 143 | -42 | 12 | 6 | | |
| Cerebellum | 75 | 2 | -54 | -24 | | |
| Occipital cortex | 47 | -14 | -78 | -30 | | |
| Occipital cortex | 28 | -34 | -64 | -26 | | |
| Cerebellum | 16 | -2 | -56 | -34 | | |
| Cerebellum | 16 | -24 | -72 | -30 | | |
| Occipital cortex, precuneus cortex | 13 | 14 | -66 | 38 | | |
| Parahippocampal gyrus | 11 | 8 | -32 | -12 | | |
| MFG, precentral gyrus | 8 | 50 | 10 | 44 | | |
| Brain stem | 5 | -4 | -40 | -22 | | |
| Frontal pole | 3 | 34 | 48 | 32 | | |
| *CS- > CS+* |  |  |  |  | | |
| Frontal pole, MFG, SFG, pre/postcentral gyrus, MTG/ITG, temporal pole, superior parietal lobule, occipital cortex, L/R hippocampus, L/R amygdala | 56455 | 46 | -50 | -18 | | |
| **Extinction** |  |  |  | |  | |
| *Late CS+E > late CS-*: no significant clusters |  |  |  |  | |  |
| *Late CS- > late CS+E*: no significant clusters |  |  |  |  | |  |
| **Extinction recall** | | | | | | |
| *Early CS+U > early CS+E*: no significant clusters |  |  |  |  | | |
| *Early CS+E > early CS+U*: no significant clusters |  |  |  |  | | |

*Note*; x, y, z values denote MNI coordinates of the peak voxel within the cluster. SFG = superior frontal gyrus, MFG = middle frontal gyrus, MTG = middle temporal gyrus, IFG = inferior frontal gyrus, ITG = inferior temporal gyrus ACC = anterior cingulate cortex, PCC = posterior cingulate cortex.

Table S5. Clusters of significant activation for extinction (CS+E vs. CS-) and associations with symptom dimensions of general distress, fears and anhedonia during late extinction (late CS+E vs. late CS-)

|  | | Voxels | X | | Y | Z | |
| --- | --- | --- | --- | --- | --- | --- | --- |
| **ACQUISITION**  **Association with General Distress** | |  |  | |  | |  |
| *CS+ > CS-*: no significant clusters | |  |  | |  | |  |
| *CS- > CS+*: no significant clusters | |  |  | |  | |  |
| **Association with Fears** | |  |  | |  | |  |
| *CS+ > CS*-: no significant clusters | |  |  | |  | |  |
| *CS- > CS+*: no significant clusters | |  |  | |  | |  |
| **Association with Anhedonia-apprehension** | | |  | |  | |  |
| *CS+ > CS-*: no significant clusters | |  |  | |  | |  |
| *CS- > CS+* | |  |  | |  | |  |
| Occipital cortex, MTG | | 817 | 40 | | -76 | | 26 |
| Occipital cortex | | 344 | -32 | | -70 | | 30 |
| Occipital cortex, posterior cingulate gyrus | | 317 | -8 | | -84 | | 18 |
| Precuneus cortex, posterior cingulate gyrus | | 15 | -21 | | -58 | | 24 |
| Occipital cortex | | 7 | -16 | | -88 | | 32 |
| Occipital cortex | | 3 | 52 | | -70 | | 3 |
| **EXTINCTION** | |  |  | |  |  | |
| **Association with General Distress** | |  |  | |  |  | |
| *Late CS+E > late CS-*: no significant clusters | |  |  | |  |  | |
| *Late CS- > late CS+E*: no significant clusters | |  |  | |  |  | |
| **Association with Fears** | |  |  | |  |  | |
| *Late CS+E > late CS-:* no significant clusters | |  |  | |  |  | |
| *Late CS- > late CS+E*: no significant clusters | |  |  | |  |  | |
| **Association with Anhedonia** | |  |  | |  |  | |
| *Late CS+E > late CS-*: no significant clusters | |  |  | |  |  | |
| *Late CS- > late CS+E* | |  |  | |  |  | |
| Frontal pole, insula, SFG, MFG, IFG, STG, MTG, ITG, anterior cingulate cortex, occipital cortex, amygdala, hippocampus, caudate, putamen | | 93207 | 32 | | -88 | -14 | |
| **RECALL**  **Association with General Distress** | | | | | | | |
| *CS+E > CS+U*: no significant clusters |  | |  |  | | |  |
| *CS+U > CS+E*: no significant clusters |  | |  |  | | |  |
| **Association with Fears** |  | |  |  | | |  |
| *CS+E > CS*+U: no significant clusters |  | |  |  | | |  |
| *CS+U > CS+E*: no significant clusters |  | |  |  | | |  |
| **Association with Anhedonia-apprehension** | | | | | | | |
| *CS+E > CS+U*: no significant clusters |  | |  |  | | |  |
| *CS+U > CS+E*: no significant clusters |  | |  |  | | |  |

*Note*: y, z values denote MNI coordinates of the peak voxel within the cluster. SFG=superior frontal gyrus, MFG=middle frontal gyrus, IFG=inferior frontal gyrus, STG = superior temporal gyrus, MTG = middle temporal gyrus, ITG = inferior temporal gyrus, CS+E=extinguished CS+; CS+U=unextinguished CS+.
